# Association of ABCC2 −24C>T Polymorphism with High-Dose Methotrexate Plasma Concentrations and Toxicities in Childhood Acute Lymphoblastic Leukemia

**DOI:** 10.1371/journal.pone.0082681

**Published:** 2014-01-03

**Authors:** Yan Liu, You Yin, Qi Sheng, Xiaotong Lu, Fang Wang, Zhiyan Lin, Huaiping Tian, Ajing Xu, Jian Zhang

**Affiliations:** 1 Department of Pharmacy, Xinhua Hospital, Shanghai Jiaotong University School of Medicine, Shanghai, China; 2 Department of Neurology, Changzheng Hospital, Second Military Medical University, Shanghai, China; 3 Department of Pediatric Hematology/Oncology, Xinhua Hospital, Shanghai Jiaotong University School of Medicine, Shanghai, China; University of Cambridge, United Kingdom

## Abstract

Methotrexate (MTX) is a key agent for the treatment of childhood acute lymphoblastic leukemia (ALL). Increased MTX plasma concentrations are associated with a higher risk of adverse drug effects. ATP-binding cassette subfamily C member 2 (ABCC2) is important for excretion of MTX and its toxic metabolite. The *ABCC2* −24C>T polymorphism (rs717620) reportedly contributes to variability of MTX kinetics. In the present study, we assessed the association between the *ABCC2* −24C>T polymorphism and methotrexate (MTX) toxicities in childhood ALL patients treated with high-dose MTX. A total of 112 Han Chinese ALL patients were treated with high-dose MTX according to the ALL-Berlin-Frankfurt-Muenster 2000 protocol. Our results showed that presence of the *−24T* allele in *ABCC2* gene led to significantly higher MTX plasma concentrations at 48 hours after the start of infusion, which would strengthen over repeated MTX infusion. The *−24T* allele in *ABCC2* gene was significantly associated with higher risks of high-grade hematologic (leucopenia, anemia, and thrombocytopenia) and non-hematologic (gastrointestinal and mucosal damage/oral mucositis) MTX toxicities. This study provides the first evidence that the *−24T* allele in *ABCC2* gene is associated with the severity of MTX toxicities, which add fresh insights into clinical application of high-dose MTX and individualization of MTX treatment.

## Introduction

Acute lymphoblastic leukemia (ALL) is the most common malignant tumor in children. The overall cure rate of ALL in children is about 80% [1]. Chemotherapy is a major element of the treatment for childhood ALL. Chemotherapy resistance is the major cause of treatment failure [1]. Methotrexate (MTX), a key agent for the treatment of childhood ALL, is a tight-binding inhibitor of the enzyme dihydrofolate reductase, which disrupts cellular folate metabolism [2]. There is a well-established relationship between MTX kinetics and toxicity [3]. High-dose MTX can significantly increase cure rates and improve patients' prognosis [4]. However, increased MTX plasma concentrations are associated with a higher risk of adverse drug effects [3]. Thus, high-dose MTX requires pharmacokinetic monitoring to avoid significant toxicities [5], and the prediction of high-dose MTX toxicity is a key issue in individualization of treatment for childhood ALL [6].

ATP-binding cassette subfamily C member 2 (ABCC2), also known as multidrug resistance-related protein 2 or canalicular multispecific organic anion transporter, is a multispecific organic anion efflux transporter that affects biliary excretion of a wide variety of endogenous and xenobiotic compounds, including doxorubicin, MTX, SN-38, and food-derived carcinogen 2-amino-1-methyl-6-phen [7]–[9]. Particularly, ABCC2 can transport MTX and its metabolites from intracellular spaces, which is important for biliary excretion of MTX and its toxic metabolite, 7-hydroxymethotrexate [10]. The *ABCC2* gene comprises 32 exons spanning 69 kb in human chromosome 10q24. Single nucleotide polymorphism (SNP) rs717620 (−24C>T) is located in the 5′ untranslated region (UTR) of the *ABCC2* gene [11]. A previous study suggests that the *ABCC2* −24C>T polymorphism contributes to variability of MTX kinetics [12]. We hypothesized that the *ABCC2* gene −24C>T polymorphism would affect the plasma concentrations of MTX and therefore its toxicities. In the present study, we explored effects of the *ABCC2* −24C>T polymorphism on MTX toxicities in childhood ALL patients treated with high-dose MTX.

## Materials and Methods

### Ethics statement

This study was approved by the Ethics Committee of Xinhua Hospital, Shanghai Jiaotong University School of Medicine. Written informed consent was obtained from the parent or guardian of each participant before the start of the study.

### Patients

Between March 2007 and June 2010, a total of 112 consecutive Han Chinese children with medium- to high-risk ALL [13] (age range, 1–10 years; mean age, 6.16±3.09 years; gender, 59 males, 53 females) were recruited to this study. Patients with liver or renal dysfunction, or taking non-steroidal anti-inflammatory drugs, probenecid, penicillin, or proton pump inhibitors were excluded.

### Treatment

According to the ALL-Berlin-Frankfurt-Muenster (BFM) 2000 protocol [12], all patients received four cycles of high-dose MTX at 5000 mg/m^2^ body surface area. One-tenth of the dose was applied through rapid infusion over 30 min, and the remainder through continuous infusion over 24 h. Leucovorin rescue (15 mg/m^2^) was administered every 6 h, starting at 48 h after initiation of MTX infusion. The patients received intravenous hydration and sodium bicarbonate according to standardized protocols to keep them well hydrated and the urine pH high [12].

### PCR and DNA sequencing

Besides the *ABCC2* −24C>T polymorphism (rs717620), rs3740065 in *ABCC2*, and rs9516519, rs868853 and rs2274407 in *ABCC4* reportedly are associated with the MTX plasma concentration and toxicities in childhood ALL [14], [15]. In addition, rs2231137 in ATP-binding cassette subfamily G member 2 (*ABCG2*) reportedly contributes to differential survival outcomes and toxicities in acute myeloid leukemia (AML) patients [16]. All the SNPs were selected for genotyping. Genomic DNA was extracted from 2 mL peripheral blood using the phenol-chloroform method as described previously [14]. For each sample, 400 ng of DNA were genotyped using the GoldenGate genotyping assay (Illumina Inc., San Diego, California, USA) with Veracode technology according to the published Illumina protocol. Data were analyzed using the GenomeStudio software (Illumina Inc.). Quality control was performed by sequencing in 60 subjects randomly selected from the 112 patients. The genotyping accuracy was 100%.

### Determination of MTX plasma concentrations and toxicities

The MTX plasma concentrations were determined by polarization fluorescence immunoassay (Viva-E/V-T, Siemens, Germany). The concentrations at 48 h after initiation of MTX infusion were used for the analysis. Toxicity data obtained from the patient files included side effects experienced during and after MTX infusion. Hematologic (leucopenia, anemia, and thrombocytopenia) and non-hematologic (hepatic, gastrointestinal, and mucosal damage/oral mucositis, etc.) toxicities were graded according to Common Terminology Criteria for Adverse Events, version 3.0 (Table S1) [17].

### Statistical analysis

Statistical analyses were performed using SPSS for Windows version 15.0 (SPSS Inc., Chicago, IL, USA). All values were expressed as mean±SD. Repeatedly measured MTX plasma concentrations were analyzed with repeated measures ANOVA. Categorical variables were compared with Chi-square tests or Fisher's exact tests. Hardy-Weinberg equilibrium analysis for genotype distribution was carried out by a Chi-square goodness-of-fit test. *p*<0.05 was considered statistically significant.

## Results

Patient characteristics are shown in Table 1. As shown in Table 2, in a total of 112 childhood ALL patients, distribution of the *ABCC2* −24C>T polymorphism (rs717620) genotypes were as follows: CC (wild type), n = 38 (33.9%); CT (heterozygous), n = 66 (58.9%); TT (homozygous), n = 8 (7.2%). The T allele frequency was 0.37. The −24C>T genotype frequencies were in Hardy-Weinberg equilibrium in the patients. According to the ALL-BFM 2000 protocol [12], all patients received four cycles of high-dose MTX at 5000 mg/m^2^ body surface area. MTX plasma concentrations were measured at 48 h after initiation of MTX infusion in each cycle. Thus, a total of 448 MTX plasma concentrations were collected.

**Table 1:** Characteristics of patients.

Total patients	112
**Continuous variables**	Mean ±SD
Age (yr)	6.16±3.09
Weight (kg)	26.31±18.57
Height (cm)	109.41±33.84
Body surface area (m^2^)	0.86±0.33
**Categorical variables**	N (%)
*Gender*	
Male	59 (52.7%)
Female	53 (47.3%)
*Liver or spleen enlargement*	59 (52.7%)
*CNS involvement*	13 (11.6%)
*Immunotyping*	
B-cell ALL	61 (54.5%)
T-cell ALL	51 (45.5%)

**Note**: CNS, central nervous system.

**Table 2:** Genotypes of selected single nucleotide polymorphisms in the patients.

ABCC2 rs717620		ABCC4 rs9516519	
*Genotype*		*Genotype*	
TT	8 (7.2%)	GG	0
TC	66 (58.9%)	GT	6 (5.4%)
CC	38 (33.9%)	TT	106 (94.6)
*Minor allele frequency*	0.37	*Minor allele frequency*	0.03
ABCC2 rs3740065		ABCC4 rs868853	
*Genotype*		*Genotype*	
CC	14 (12.5%)	CC	0
CT	43 (38.4%)	CT	27 (24.1)
TT	55 (49.1%)	TT	85 (75.9)
*Minor allele frequency*	0.32	*Minor allele frequency*	0.12
ABCG2 rs2231137		ABCC4 rs2274407	
*Genotype*		*Genotype*	
AA	0	AA	3 (2.7%)
AG	69 (61.6%)	AC	33 (29.5%)
GG	43 (38.4%)	CC	76 (67.8%)
*Minor allele frequency*	0.31	*Minor allele frequency*	0.17

**Note**: ABCC2, ATP-binding cassette subfamily C member 2; ABCC4, ATP-binding cassette subfamily C member 4; ABCG2, ATP-binding cassette subfamily G member 2.

**Figure 1:**
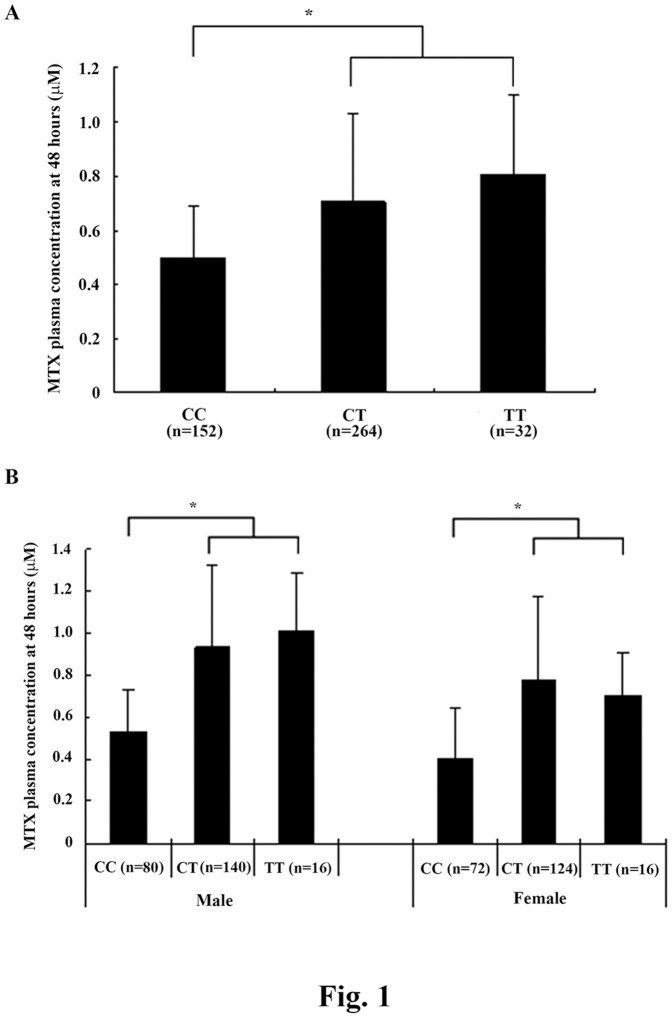
Methotrexate (MTX) plasma concentrations by ATP-binding cassette subfamily C member 2 (*ABCC2*) −24C>T polymorphism genotypes. MTX plasma concentrations were measured at 48*ABCC2* −24C>T polymorphism genotypes alone (*A*) or further stratified by gender (*B*). *p<0.05 vs CC.

**Figure 2:**
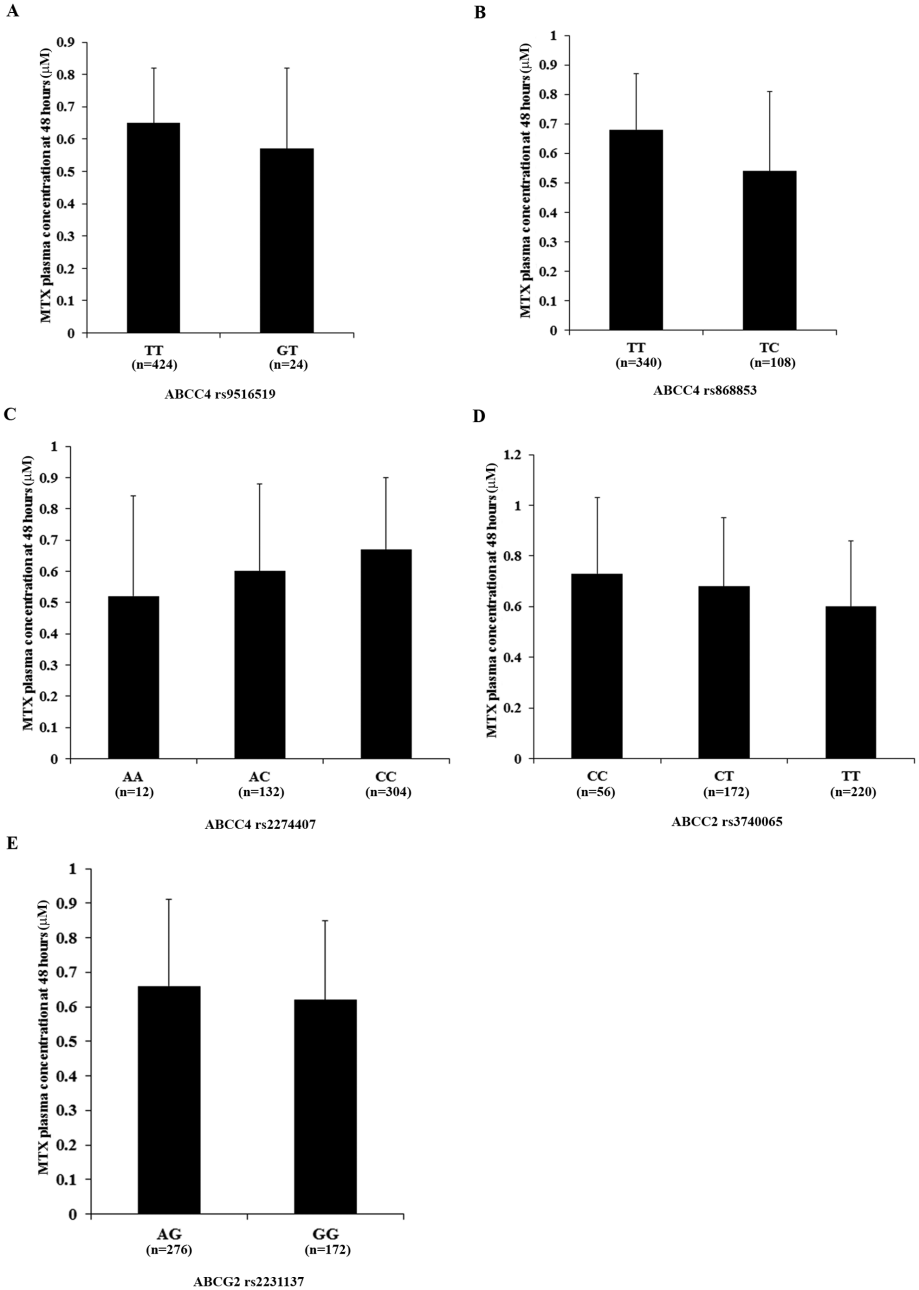
Methotrexate (MTX) plasma concentrations by single nucleotide polymorphism (SNP) genotypes of ATP-binding cassette subfamily C member 2 (*ABCC2), ABCC4 and ABCG2*. MTX plasma concentrations were measured at 48*ABCC4* rs9516519 (*A*), *ABCC4* rs868853 (*B*), *ABCC4* rs2274407 (*C*), *ABCC2* rs3740065 (*D*), *ABCG2* rs2231137 (*E*).

As shown in Fig. 1, at 48 h after initiation of MTX infusion, patients with at least one −24T allele (CT or TT) showed significantly higher MTX plasma concentrations than those with a wild type genotype (−24CC), without significant gender differences. In contrast, other SNPs reportedly associated with the MTX plasma concentration and toxicities in childhood ALL, including rs3740065 in *ABCC2*, and rs9516519, rs868853 and rs2274407 in *ABCC4* [14], [15], showed no significant effects on the MTX plasma concentration in this study (Fig. 2). A SNP in *ABCG2* (rs2231137) that reportedly contributes to differential survival outcomes and toxicities in AML patients showed no significant effect on the MTX plasma concentration and was used as a negative control here (Fig. 2).

As each patient had multiple measurements, repeated measures ANOVA was performed to evaluate the main effect of time and the *ABCC2* −24C>T polymorphism genotypes on MTX plasma concentrations. The effect of interaction between time and the *ABCC2* −24C>T genotypes was also assessed. F values and corresponding *p* values for each test are listed in Table 3. The partial eta squared values, which indicates the percentage of variance in MTX plasma concentrations attributable to the effect of time, the *ABCC2* −24C>T genotypes or interaction between time and the *ABCC2* −24C>T genotypes, are also listed. As shown in Tables 3, repeated measures ANOVA revealed that time had no significant main effect on the MTX plasma concentration. The *ABCC2* −24C>T genotype showed significant main effect as well as interaction with time, indicating that the *ABCC2* −24C>T polymorphism led to significant differences in MTX plasma concentrations among childhood ALL patients, and that the effect strengthened over repeated MTX infusion.

**Table 3:** Effects of ATP-binding cassette subfamily C member 2 (*ABCC2*) gene −24C>T genotype and time on methotrexate (MTX) plasma concentration.

Dependent Variable	Genotype			Time			Time×Genotype		
	F	*p*	Partial Eta Squared	F	*p*	Partial Eta Squared	F	*p*	Partial Eta Squared
MTX Plasma Concentration	20.69	0.00	0.31	1.57	0.23	0.04	8.92	0.00	0.12

**Note**: Methotrexate (MTX) plasma concentration was analyzed with repeated measures ANOVA.

In the 112 childhood ALL patients, the most common toxicities were vomiting (62.5%), mucosal damage/oral mucositis (57.1%), leucopenia (52.7%), fever (42.9%), and thrombocytopenia (36.7%). Relatively few children showed liver (13.4%) or renal (7.1%) function damage. Except for mucosal damage, no significant association was detected among *ABCC2* −24C>T genotypes and MTX toxicities (Table 4). However, after we graded the toxicities according to Common Terminology Criteria for Adverse Events version 3.0 (Table S1) [17], patients with at least one *−24T* allele (CT+TT) showed significantly higher risk for severe (grades 3–4) leucopenia (*p* = 0.02; OR = 4.74; 95% CI = 1.46–15.33), anemia (*p* = 0.02; OR = 6.11; 95% CI = 1.42–26.36), thrombocytopenia (*p* = 0.02; OR = 5.87; 95% CI = 1.40–24.67), mucosal damage/oral mucositis (*p*<0.01; OR = 9.00; 95% CI = 2.11–38.35), and gastrointestinal/vomiting (*p*<0.01; OR = 11.08; 95% CI = 3.48–35.21) than those with a wild type genotype (−24CC) (Table 5). The results indicate that the *ABCC2* −24C>T polymorphism can result in significant differences in the severity of MTX toxicities among childhood ALL patients.

**Table 4:** Incidence of methotrexate (MTX) toxicity by ATP-binding cassette subfamily C member 2 (*ABCC2*) gene −24C>T genotype.

		Incidence of MTX toxicity		
	Total patients n (%)	CC n (%)	CT+TT n (%)	*p* *
	112 (100)	38 (100)	74 (100)	
Leucopenia	59 (52.7)	19 (50.0)	40 (54.1)	0.70
Thrombocytopenia	41 (36.7)	13 (34.2)	28 (37.8)	0.84
Anemia	39 (34.8)	13 (34.2)	26 (35.1)	1.00
Fever	48 (42.9)	18 (47.4)	30 (40.5)	0.550
Mucosal damage (Oral mucositis)	64 (57.1)	12 (31.6)	52 (70.3)	<0.01
Vomiting	70 (62.5)	25 (65.8)	45 (60.8)	0.68
Diarrhea	38 (33.9)	10 (26.3)	28 (37.8)	0.29
Liver function damage	15 (13.4)	5 (13.2)	10 (13.5)	1.00
Renal function damage	8 (7.1)	3 (7.9)	5 (6.8)	1.00

**Note**: *Chi-square p value. Fisher's exact tests were performed when the toxicity (renal function damage) had expected count less than 5.

**Table 5:** Association of ATP-binding cassette subfamily C member 2 (*ABCC2*) gene −24C>T genotype with methotrexate (MTX) toxicity grades.

MTX toxicity	Genotype (n)	Toxicity grades 1–2	Toxicity grades 3–4	*p* *	OR (95% CI)
Leucopenia	CC (19)	11	8	0.02^a^	4.74 (1.46–15.33)
	CT+TT (40)	9	31		
Thrombocytopenia	CC (13)	8	5	0.02^a^	5.87 (1.40–24.67)
	CT+TT (28)	6	22		
Anemia	CC (13)	9	4	0.02^a^	6.11 (1.42–26.36)
	CT+TT (26)	7	19		
Fever	CC (18)	7	11	0.55	1.49 (0.44–5.07)
	CT+TT (30)	9	21		
Mucosal damage (Oral mucositis)	CC (12)	9	3	<0.01^b^	9.00 (2.11–38.35)
	CT+TT (52)	13	39		
Vomiting	CC (25)	19	6	<0.01^b^	11.08 (3.48–35.21)
	CT+TT (45)	10	35		
Liver function damage	CC (5)	2	3	0.56	2.67 (0.25–28.44)
	CT+TT (10)	2	8		
Renal function Damage	CC (3)	2	1	1.00	3.00 (0.15–59.89)
	CT+TT (5)	2	3		

**Note**: *Chi-square p value; Fisher's exact tests were performed when the toxicity (liver or renal function damage) had expected count less than 5.

^a^ *p*<0.05;

b *p*<0.01.

## Discussion

In the present study, we explored effects of the *ABCC2* −24C>T polymorphism (rs717620) on MTX plasma concentrations in a relatively large childhood ALL patient population (n = 112) treated with high-dose MTX, and for the first time assessed the association between the *ABCC2* −24C>T polymorphism and MTX toxicities.

High-dose MTX forms a cornerstone in the treatment of several pediatric malignancies including ALL. The *ABCC2* gene is involved in active efflux of MTX [18], [19]. The *ABCC2* −24C>T polymorphism reportedly contributes to variability of MTX kinetics [12]. Rau et al. reported that the mean plasma MTX area under the curve from 36 to 48 hours after the start of infusion was significantly higher in female patients carrying at least one −*24T* allele compared with other patients [12]. In agreement with this report, patients with at least one *−24T* allele showed significantly higher MTX plasma concentrations than those with a wild type genotype (−24CC) in our study. However, no significant difference between genders was noted. Simon et al. reported that MTX clearance and distribution volume were significantly higher in carriers of at least one copy of the *−24T* allele as compared with non-carriers [3], suggesting that the *−24T* allele could lead to lower MTX plasma concentrations. In addition, Ramsey et al. reported that male childhood ALL patients had greater MTX clearance [20]. The discrepancies between previous studies and our study were probably due to different study populations, sample sizes and treatment protocols used: Rau's study was conducted with 44 German childhood ALL patients following the ALL-BFM 95 or 2000 protocol (high-dose MTX 5000 mg/m^2^ in a 30 min-24 h i.v. infusion) [12], our study conducted with 112 Chinese childhood ALL patients following the ALL-BFM 2000 protocol (high-dose MTX 5000 mg/m^2^ in a 30 min-24 h i.v. infusion), Simon's study conducted with 50 French adult patients with large-cell lymphoma, Burkitt's lymphoma or ALL using MTX 1000 to 8000 mg/m^2^ in a 1–6 h i.v. infusion [3], and Ramsey's study conducted with 1279 childhood ALL patients with various ethnic backgrounds (including 806 Caucasians, 266 Hispanics, 58 blacks, and only 22 Asians) on four different MTX regimens [20].

Besides the *ABCC2* −24C>T polymorphism (rs717620), other SNPs reportedly associated with the MTX plasma concentration and toxicities in childhood ALL, including rs3740065 in *ABCC2*, and rs9516519, rs868853 and rs2274407 in *ABCC4* [14], [15], were examined in this study and showed no significant effects on the MTX plasma concentration. This is very likely due to ethnic differences in the study populations, for our study has been the only one carried out in Han Chinese, which accounts for 90% of the population in China and 19% of global population [21].

Our results showed that presence of the *−24T* allele in *ABCC2* gene led to significantly higher MTX plasma concentrations in childhood ALL patients, which would strengthen over repeated MTX infusion. MTX kinetics is well related to its efficacy and toxicity, and increased plasma concentrations are associated with a higher risk of adverse drug effects [3]. Indeed, we found that the *−24T* allele in *ABCC2* was significantly associated with higher risks of high-grade hematologic (leucopenia, anemia, and thrombocytopenia) and non-hematologic (gastrointestinal and mucosal damage/oral mucositis) MTX toxicities in childhood ALL patients. Thus, the *ABCC2* −24C>T polymorphism could be used to predict MTX toxicities and guide individualized treatment for childhood ALL, since prediction of MTX toxicities is a key issue in individualization of MTX treatment [6]. The −*24T* allele frequency in *ABCC2* was 0.37 in Chinese childhood ALL patients, and 0.16 in Caucasians [3], suggesting that there are significant ethnic differences in distribution of the *ABCC2* −24C>T genotypes. Therefore, the effect of the *ABCC2* −24C>T polymorphism on MTX plasma concentrations and toxicities is very likely ethnicity-specific.

The molecular mechanism how the *ABCC2* −24C>T polymorphism regulates the MTX plasma concentration remains unclear and elusive. The −24C>T polymorphism is located in the 5′ UTR of *ABCC2* gene [11]. Zhang et al. reported that characterization of the *ABCC2* −24C>T polymorphism demonstrated no effect on mRNA expression or downstream open reading frame translation, minimizing the possibility of the SNP's involvement in transcriptional and translational regulation of ABCC2 expression, which was confirmed in our pilot studies (data not shown). It will be intriguing to uncover mechanisms underlying the effects of *ABCC2* −24C>T on the MTX plasma concentration in future studies.

In conclusion, we demonstrate in Han Chinese childhood ALL patients that MTX plasma concentrations and toxicity severity are significantly higher in carriers of at least one copy of the *−24T* allele in *ABCC2* gene compared with non-carriers. This study provides the first evidence that the *−24T* allele in *ABCC2* gene is associated with the severity of MTX toxicities, which add fresh insights into clinical application of high-dose MTX and individualization of MTX treatment, particularly, for childhood ALL.

## Supporting Information

Table S1 **Common Terminology Criteria for Adverse Events version 3.0.**
(DOC)
