# Correction: Association of ABCC2 -24C>T Polymorphism with High-Dose Methotrexate Plasma Concentrations and Toxicities in Childhood Acute Lymphoblastic Leukemia

**DOI:** 10.1371/journal.pone.0091384

**Published:** 2014-03-21

**Authors:** The PLOS ONE staff

There are multiple errors in the Funding statement. The Funding statement should read: "This work was supported by Natural Science Foundation of China (NSFC) (grant #81202598), Shanghai Municipal Public Health Bureau (grant #2009068), and Science and Technology Commission of Shanghai Municipality (grants #11DZ1972500 and #12DZ1930404). The funders had no role in study design, data collection and analysis, decision to publish, or preparation of the manuscript.”
