# Supplementary material for: Association of ABCC2 −24C>T Polymorphism with High-Dose Methotrexate Plasma Concentrations and Toxicities in Childhood Acute Lymphoblastic Leukemia
Source: PLoS One. 2014 Jan 3;9(1):e82681. doi: 10.1371/journal.pone.0082681 (PMC3880259; doi:10.1371/journal.pone.0082681)
Supplement: Table S1 — Common Terminology Criteria for Adverse Events version 3.0. (DOC) [file pone.0082681.s001.doc]

**Table S1. Common Terminology Criteria for Adverse Events version 3.0**

| **TOXICITY** | | **Grade** | | | | |
| --- | --- | --- | --- | --- | --- | --- |
|  |  | **0** | **Ⅰ** | **Ⅱ** | **Ⅲ** | **Ⅳ** |
| **Hematologic damage** | | | | | | |
| Anemia | Hemoglobin（g/L） | ≥110 | 95~109 | 80~94 | 65~79 | <65 |
| Leukopenia | Leucocyte（×109/L） | ≥4.0 | 3.0~3.9 | 2.0~2.9 | 1.0~1.9 | <1.0 |
| Thrombocytopenia | Thrombocyte（×109/L） | ≥100 | 75~99 | 50~74 | 25~49 | <25 |
| **Liver function damage** | | | | | | |
| bilirubin | | ≤1.25×N | 1.26~2.50×N | 2.6~5.0×N | 5.1~10.0×N | >10×N |
| AST | | ≤1.25×N | 1.26~2.50×N | 2.6~5.0×N | 5.1~10.0×N | >10×N |
| ALP | | ≤1.25×N | 1.26~2.50×N | 2.6~5.0×N | 5.1~10.0×N | >10×N |
| **Mucosal damage** | | Normal | Erythema | Ulcer, can eating | Ulcer, just can eat liquid diet | Can not eat |
| **Vomiting** | | Normal | 1 episode in 24 hrs | 2 – 5 episodes in 24 hrs;IV fluids indicated <24 hrs | ≥6 episodes in 24 hrs; IV fluids, or TPN indicated ≥24 hrs | Life-threatening consequences |
| **Renal function damage** | | | | | | |
| BUN | | ≤1.25×N | 1.26~2.50×N | 2.6~5.0×N | 5.1~10.0×N | >10×N |
| creatinine | | ≤1.25×N | 1.26~2.50×N | 2.6~5.0×N | 5.1~10.0×N | >10×N |
|  | |  |  |  |  |  |
| **Allergic reaction/**  **hypersensitivity**  **(including drug fever)** | | Allergic reaction | Transient flushing or rash; drug fever <38°C(<100.4°F) | Rash; flushing; urticaria;  dyspnea; drug fever≥38°C (≥100.4°F) | bronchospasm, with or  without urticaria;  parenteral medication(s)  indicated; allergy-related  edema/angioedema;hypotension | Anaphylaxis |
